# Lactic acid bacteria isolated from dairy products as potential producers of lipolytic, proteolytic and antibacterial proteins

**DOI:** 10.1007/s00253-019-09844-6

**Published:** 2019-04-27

**Authors:** Israel García-Cano, Diana Rocha-Mendoza, Joana Ortega-Anaya, Karen Wang, Erica Kosmerl, Rafael Jiménez-Flores

**Affiliations:** Department of Food Science and Technology, Parker Food Science and Technology Building, The Ohio State University, Columbus, OH 43210 USA

**Keywords:** Lactic acid bacteria, Dairy products, Zymography, Bioactivity

## Abstract

Regular consumption of fermented dairy products helps maintain a healthy microbiota and prevent gut dysbiosis-linked diseases. The lactic acid bacteria (LAB) present in food enhance the digestibility of proteins, moderate the release of fatty acids, and support human health through inhabiting the gastrointestinal tract. These desirable properties of LAB are attributed, in part, to their metabolic processes involving enzymes such as lipases, proteases, and antibacterial proteins. The LAB strains presenting higher enzymatic activities may offer improved functionality for applications in foods. The first aim of this work was to isolate and identify LAB from diverse dairy products and select those with enhanced enzymatic activities. Secondly, this work aimed to investigate the subcellular organization and identity of these enzymes after semi-purification. Out of the total 137 LAB strains isolated and screened, 50.3% and 61.3% of the strains exhibited lipolytic and proteolytic activities, respectively. Seven strains displaying high enzymatic activities were selected and further characterized for the cellular organization of their lipases, proteases, and antibacterial proteins. The lipolytic and proteolytic activities were exhibited predominantly in the extracellular fraction; whereas, the antibacterial activities were found in various cellular fractions and were capable of inhibiting common undesirable microorganisms in foods. In total, two lipases, seven proteases, and three antibacterial proteins were identified by LC-MS/MS. Characterization of LAB strains with high enzymatic activity has potential biotechnological significance in fermentative processes and in human health as they may improve the physicochemical characteristics of foods and displace strains with weaker enzymatic activities in the human gut microbiota.

## Introduction

Lactic acid bacteria (LAB) encompass a wide group of microorganisms belonging to the genera *Enterococcus*, *Lactobacillus*, *Lactococcus*, *Leuconostoc*, *Pediococcus*, and *Streptococcus*, among others (Burgain et al. 2014). LAB, found naturally in some foods, participate in food fermentation processes and are intentionally added as starter cultures to improve color development, reduce ripening periods, and enhance sensory characteristics including flavor, aroma, and texture. Additionally, LAB fermentation improves food digestibility and increases product safety through inhibition of spoilage and pathogenic bacteria (Papagianni 2012). Beyond these important physicochemical roles, some LAB strains are considered to be probiotics due to their beneficial effect on the intestinal tract and human health (Jankovic et al. 2010). These meaningful implications make understanding the functionality of LAB an industrially and nutritionally relevant topic.

The biochemistry of fermented products is complex, involving a series of many enzymatic reactions. Among the changes that occur during food fermentation are a decrease in pH and the inhibition of other microorganisms due to the production of metabolites such as lactic acid, hydrogen peroxide, diacetyl, acetaldehyde, reuterin, and peptides (Papagianni 2012). Additionally, fermentation leads to the production of antibacterial compounds and other molecules derived from metabolism such as lipolytic and proteolytic enzymes (Burgain et al. 2014).

Lipolysis is the hydrolysis of triglycerides to produce free fatty acids, glycerol, and intermediates such as mono- and diglycerides. These intermediates emulsify other food components, which aid in texture development of the final product (Esteban-Torres et al. 2014). The degree of lipolysis by LAB helps determine the selection of the strains used as starter cultures. This property is extremely relevant for vegetable fermentation, baked food applications, and flavor development in fermented milk products (Tanasupawat et al. 2015).

LAB are generally considered to be weakly lipolytic relative to species of the *Penicillium* and *Pseudomonas* genera (McSweeney and Sousa 2000). However, in products such as cheeses with extended ripening periods, lipolytic activity from LAB contribute to flavor development and serve as substrates for further reactions producing catabolic end products (Collins et al. 2003).

LAB are not distinguished for their lipolysis, but some reports of lipolytic activity have been made in *Lactobacilli* strains. Specifically, the lipolytic activity of *L. helveticus*, *L. delbrueckii*, *L. bulgaricus. L. casei*, *L. plantarum*, and *L. acidophilus* species have been studied (El-Soda et al. 1986; Fernandez et al. 2000).

Proteolysis is the hydrolysis of peptide bonds in proteins to generate peptides and free amino acids. Free amino acid formation and subsequent participation in decarboxylation, deamination, transamination, and desulfurization reactions play a crucial role in determining food flavor. Proteases are pertinent to the development of fermented products because they are associated with the release of these molecules directly responsible for desirable aroma, texture modification, and reduced a_w_ of some products such as aged cheeses (Savijoki et al. 2006). In fermented dairy foods, milk protein proteolysis improves the digestibility of products, a characteristic highly desired by the infant formula industry. Proteolysis also reduces whey protein antigenicity thought to provoke an immune response in some individuals. Furthermore, the bioactive peptides obtained from milk protein proteolysis reduce microbial infections (Atanasova et al. 2014). Many LAB including *Lactococcus*, *Lactobacillus*, and *Streptococcus* are considered weakly proteolytic strains. Nevertheless, proteolysis is one of the major and more complex enzymatic reactions occurring in dairy products (McSweeney and Sousa 2000). LAB possess a very intricate proteinase/peptidase system which is comprised of three components: proteases bound to the cell wall that initiate the degradation of casein into oligopeptides, peptide transporters, and intracellular peptidases that degrade peptides into shorter peptides and free amino acids. The components of the LAB proteolytic system have been studied by genome analysis; however, the complexity of this system hinders the purification and identification of many involved proteins that remain uncovered (Liu et al. 2010).

Lastly, the proteins exerting antibacterial activity have been widely studied. Among these, bacteriocins constitute one of the most important classes of antibacterial proteins found in LAB. Bacteriocin is a generic term for peptides with antimicrobial activity. Bacteriocins are a result of adaptation that has allowed LAB to compete favorably against other phylogenetically related microorganisms for the nutrients in their habitat and participate in cellular communication (Álvarez-Sieiro et al. 2016). They have been classified and categorized by several ways: molecular weight, sequence or post-translational modifications, source organism, lytic ability, mode of action, and substrate or target, among others. Regardless of these classifications, bacteriocins that degrade peptidoglycan in the bacterial cell wall are designated as bacterial cell wall hydrolases, autolysins, or peptidoglycan hydrolases (PGHs) (Callewaert et al. 2011). These enzymes exert their antibacterial activity by hydrolysis of the glycosidic or peptide bonds of peptidoglycan, the major component of the bacterial cell walls. Bacteriocins in general and specifically PGHs from LAB show potential for application in GRAS biopreservation of fermented food since they have potent activity in vivo and in vitro especially against pathogenic Gram-positive bacteria. Moreover, these proteins have activity against antibiotic-resistant bacteria because of their unique mechanism of action, completely different from that of antibiotics (Callewaert et al. 2011; Rodríguez-Rubio et al. 2016). Because of the wide variety of enzymes with antimicrobial activity produced by LAB, the discovery and characterization of new or putative proteins constitutes a niche of research that is imperative to explore.

As mentioned, LAB constitute an important source of enzymes that help shape desired sensory characteristics in dairy products and impart benefits on the gastrointestinal tract when ingested in the diet. The aim of this work was to investigate the enzymatic activities of LAB isolated from dairy products and determine their subcellular organization and identity after semi-purification. This study is of significance as it provides insight into LAB metabolism of food-derived components and has a potential impact in modification of the microbiome upon ingestion, as shown in many in vitro and in vivo studies (Derrien and van Hylckama Vlieg 2015).

## Materials and methods

### Bacterial isolation

Dairy products including raw milk, milk powder, buttermilk powder, yogurt, cheese, and cream were purchased from local food stores (Columbus, OH, USA). Twenty-five grams of each sample was homogenized in 250 ml of sterile saline solution (0.85% NaCl, pH 7.0) using a Stomacher 80 Biomaster for 1 min at 300 rpm (High speed, Seward, UK). Afterwards, serial dilutions of 10 ml were prepared from each sample and plated on MRS agar (Difco, USA) with bromocresol green (0.0025%) as a pH indicator. The plates were incubated for 24–48 h at 37 °C under anaerobic conditions. Colonies were selected based on phenotypic features such circular shape, creamy texture, white, gray, or green color and were further regrown in MRS broth and agar until complete isolation.

### Screening of lipolytic and proteolytic activity

Lipolytic activity screening was performed following the protocol described by Peña-Montes et al. (2013) using *p*-nitrophenyl acetate as substrate (*p*-NPA; Sigma-Aldrich, USA). Fifty microliter of each isolated strain culture were mixed with 50 μl of substrate and incubated at 37 °C for 1 min intervals over a 1 h. The absorbance of the colored complex was measured at 410 nm in a spectrophotometer (Multiskan Go, Thermo Fisher, USA). Lipase from porcine pancreas (Sigma-Aldrich) was used as positive control. One unit was defined as the quantity of enzyme that releases 1 μmol of *p*-nitrophenol per min at 37 °C. The proteolytic activity was determined by quantification of colorimetric azo groups released from a synthetic azocasein (Sigma-Aldrich), as Bendicho et al. (2002) described. Fifty microliter of each strain culture were mixed with 500 μl of azocasein (0.5 mg/ml in Tris-HCl, pH 7.6) and incubated at 37 °C for 2 h. The reaction was stopped with 500 μl of 10% TCA (Fisher Chemical, USA). The suspension was centrifuged at 13,000×*g* for 10 min. One-part supernatant was mixed with one-part 50% NaOH. α-chymotrypsin from bovine pancreas (Sigma-Aldrich) was used as a positive control. The absorbance of azo groups was measured at 440 nm and the final proteolytic activity was defined as the amount of the enzyme yielding *Δ*Abs_440nm_ = 0.01 per min.

*Lactococcus lactis* strain purchased from Chr. Hansen (Hoersholm, Denmark) was used as a positive control. The strain was inoculated in MRS broth and incubated at 37 °C for 12 h, and then the culture was used for lipolytic and proteolytic activities.

### Antibacterial activity

To analyze the antibacterial activity towards different substrates, four techniques were employed: (a) N-acetylglucosaminidase activity was measured using the substrate 4-nitrophenyl N-acetyl-β-D-glucosamine (NP-GlcNAc; Sigma-Aldrich; García-Cano et al. 2015). Ten microliter of 1 mg/ml NP-GlcNAc solution was placed in a 96-well plate along with 10 μl of sample and 80 μl of sodium citrate buffer (100 mM, pH 4.8). Ten microgram per milliliter of the β-nacetylglucosaminidase from *Canavalia ensiformis* (Sigma-Aldrich) was used as a positive control. The reaction mixture was incubated at 37 °C for 10 min and 100 μl of sodium carbonate (140 mM) was used to stop the reaction and enhance the color. Absorbance at 405 nm was determined. One unit was defined as the amount of enzyme that hydrolyzes 1.0 μmol of 4-nitrophenyl N-acetyl-β-D-glucosamine to 4-nitrophenol and N-acetyl-β-D-glucosamine per minute at pH 4.7 at 37 °C. (b) N-acetylmuramoyl-L-alanine amidase activity was performed using the substrate L-alanine-*p*-nitroanilide hydrochloride (Sigma-Aldrich; García-Cano et al. 2015). Ten microliter of 1 mg/ml substrate solution was placed in a 96-well plate along with 10 μl of the assayed sample and 80 μl Tris-HCl buffer (100 mM, pH 7.6). The reaction mixture was incubated at 37 °C for 10 min. Absorbance at 405 nm was determined. One unit was defined as the amount of enzyme that will hydrolyze 1.0 μmol of L-alanine-*p*-nitroanilide to *p*-nitroaniline per minute at pH 7.6 at 37 °C. An amidase from *Pseudomonas aeruginosa* (Sigma-Aldrich) was used as a positive control. (c) The turbidimetric assay for *N*-acetylmuramidase activity was measured using whole *Micrococcus lysodeikticus* cells (Sigma-Aldrich; Morsky 1983). The cells were resuspended in Tris-HCl pH 7.6 until an optical density (O.D._450nm_) of 1.0 was obtained. Two hundred microliter of each suspension was preincubated at 37 °C for 10 min. The reaction was initiated by the addition of 50 μl of the sample. The reaction mixture was measured at 37 °C for 1 min intervals over a 1 h. One unit of lytic activity was defined as the amount of enzyme resulting in a decrease in the *Δ*O.D._450nm_ of 0.001 per min. Lysozyme from chicken egg white (Sigma-Aldrich) was used as a positive control. (d) Endopeptidase activity was performed using peptidoglycan from *Staphylococcus aureus* as the substrate in the turbidimetric assay (Sigma-Aldrich; Zhou et al. 1988). Peptidoglycan was resuspended in Tris-HCl pH 7.6 until O.D._620nm_ reached 0.5. Two hundred microliter of each suspension was preincubated at 37 °C for 10 min. The reaction was initiated by the addition of 50 μl of the sample. The reaction mixture was measured at 37 °C for 2 min intervals over a 2 h. One unit of lytic activity was defined as the amount of enzyme resulting in a decrease in the *Δ*O.D._620nm_ of 0.001 per min. Lysostaphin from *Staphylococcus staphylolyticus* (Sigma-Aldrich) was used as a positive control. To complement these experiments, agar diffusion assays were performed as described by García-Cano et al. (2014) using the following strains as targets: *Escherichia coli* ATCC 25922, *Listeria innocua* ATCC 51742, *Staphylococcus aureus* ATCC 25923, and *Staphylococcus epidermidis* ATCC 1222. All strains were grown in BHI broth (Difco, USA) at 37 °C for 12–14 h without stirring.

### Identification of bacterial strains by 16S rRNA sequencing

Genomic DNA (gDNA) was extracted from freshly prepared cultures grown in MRS broth. The cells were first pelleted by centrifugation at 10,000×*g* for 10 min at 4 °C (Centrifuge 5415R, Eppendorf, USA) and washed two times using one volume of sterile saline solution. The gDNA of each LAB was isolated using Wizard Genomic DNA Purification Kit (Promega, USA) following the manufacturer’s protocol. To amplify the complete 16S rRNA gene, identical conditions and primers reported by García-Cano et al. (2014) were used in conjunction with a Platinum Taq DNA Polymerase (Thermo Scientific, USA) in a Thermal Cycler Eco (Eppendorf, Germany). The resulting amplicons were purified using a Wizard® SV Gel and PCR Clean-Up system (Promega, USA), and the sequencing experiments were performed by Macrogen Inc. (Seoul, South Korea). The sequences obtained were aligned with the 16S rRNA reference gene sequence from GenBank using the Basic Local Alignment Search Tool (BLAST) algorithm available from the National Center of Biotechnology Information (date of query: May 2018).

### Accession number

The 16S rRNA sequences have been deposited at GenBank under the accession number: *P. acidilactici* OSU-PECh-L, MK543955; *L. casei* OSU-PECh-C, MK543956; *L. plantarum* OSU-PECh-A, MK543957; *P. acidilactici* OSU-PECh-3A, MK543958; *L. paracasei* OSU-PECh-3B, MK543959; *L. paracasei* OSU-PECh-BA, MK543960; and *L. plantarum* OSU-PECh-BA, MK543961.

### Preparation of cellular fractions

In order to identify the cellular location of the aforementioned enzymes, four cell fractions were prepared and evaluated, as follows: (a) cell-free extract (supernatant), (b) intracellular fraction, (c) cell-debris fraction, and (d) whole cell fraction. After overnight incubation at 37 °C, the whole culture media was centrifuged at 8000×*g* for 10 min at 4 °C, and the supernatant fraction was removed from the bacterial cell pellet. After the pH was adjusted to 7.0, the supernatant fraction was filtered by a 0.22 μm cutoff sterile membrane (Millipore, USA) and kept at − 20 °C. The pellet, corresponding to the whole cell fraction, was washed two times with one volume of PBS and resuspended in the same buffer before adjusting the O.D._600nm_ to 2.5 in order to standardize the enzymatic activities. The intracellular and cell-debris fractions were obtained using ultrasonication to disrupt the cells by the following conditions: 20 cycles of 20 s sonication at 20 Hz followed by 20 s rest (Branson Sonifier 450; Fisher, USA). The intracellular fraction was recovered by centrifugation at 15,000×*g* for 30 min at 4 °C, and the cell-debris fraction (pellet) was also collected. The four factions obtained were tested for enzymatic activity.

### Protein concentration assay

The amount of protein in each fraction was measured following the instructions of Bradford protein assay kit (Bio-Rad, USA). Bovine serum albumin was used as a protein standard.

### Protein isolation by zymography

To isolate the proteins responsible for each enzymatic activity, zymography was performed with copolymerized polyacrylamide gels testing each cell fraction obtained. Briefly, lipolytic activity was observed by 10% polyacrylamide gel electrophoresis (PAGE) using α-naphthyl acetate as the substrate (Sigma-Aldrich), according to Peña-Montes et al. (2013). The proteolytic activity was tested with SDS-PAGE using casein as the substrate, as described by Stuknytė et al. (2016). The antibacterial activity against *M. lysodeikticus* was evaluated according to García-Cano et al. (2015). The molecular weight of the bands displaying lipolytic, proteolytic, or lytic activities were determined using a Precision Plus Protein™ Standard (Bio-Rad, USA) and analyzed using a ChemiDoc™Touch imaging system (Bio-Rad, USA).

Each protein band that displayed activity in the zymograms was excised from the gel, placed in a 1.5 mL tube, triturated in 500 μL of sterile deionized H_2_O, and incubated overnight at room temperature. Afterwards, the solution was filtered through a 0.22 μm cutoff sterile membrane to eliminate the gel material and was subsequently concentrated by ultrafiltration with an Amicon® Ultra Centrifugal Filter (0.5 ml, 10-kDa cutoff; Millipore, USA). Finally, each isolated protein was loaded into an SDS-PAGE for further analysis by LC-MS/MS.

### Protein identification by LC-MS/MS

The proteins were sent for sequencing analysis at the Campus Chemical Instrument Center (CCIC), Mass Spectrometry and Proteomics Facility at The Ohio State University (Columbus, Ohio, USA). Protein bands were subjected to in-gel trypsin digestion, and the resulting peptides analyzed by capillary LC-MS/MS. Peptide fragments generated by tandem MS were compared with the MASCOT database to obtain the amino acid sequence. Proteins with at least two matching peptide fragments to the database were considered reliable identification hits.

## Results

### Bacteria isolation, initial screening of lipolytic and proteolytic activities, and strain identification by 16S rRNA sequencing

**Fig. 1:**
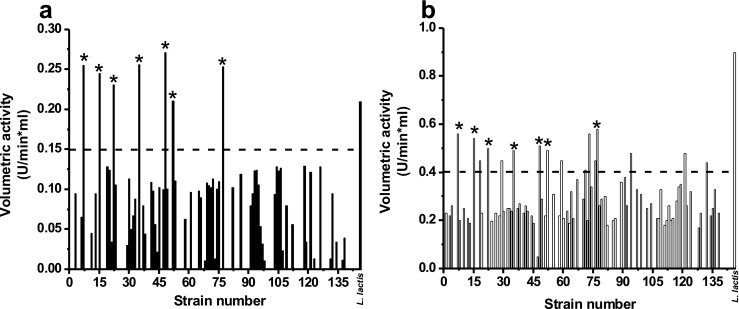
Screening of **a** lipolytic and **b** proteolytic activities of 137 strains isolated from different dairy products. *corresponds to the selected strains. --- represents the cutoff for selection. *L*. *lactis* was used as a positive control

After initial selection, each strain was grown in MRS agar plates to confirm isolation. A total of 137 strains were isolated from different dairy products and given a specific designation code (OSU-PECh). The initial screening for lipolytic activity, using *p*-NPA as the substrate, showed that 50.3% of the total isolated strains exhibited lipolytic activity and, furthermore, seven strains resulted in high lipolysis compared to the positive control (*L. lactis*; Fig. 1a). Values above 0.15 U/min*mL were deemed highly lipolytic. Conversely, screening for proteolytic activity using azocasein as a substrate showed that 61.3% of the strains displayed proteolytic activity and 15 of them had values above 0.4 U/min*mL, which were considered highly proteolytic strains (Fig. 1b). However, the positive control (*L. lactis)* proteolytic activity was 0.87 U/min*mL, which was about two times higher than the isolated strains in this study.

Based on this screening, seven strains displayed both high lipolytic and proteolytic activity and were further subjected to gDNA extraction and identification by 16S rRNA gene sequencing. These strains corresponded to the following species isolated from commercial dairy products: one strain of *Lactobacillus casei* (*L. casei* OSU-PECh-C) isolated from whey protein isolate produced by Hilmar Ingredients (Hilmar CA, USA); two strains of *Lactobacillus paracasei* (*L. paracasei* OSU-PECh-BA) isolated from milk protein concentrate produced by Idaho Milk Products (Salt Lake City, UT, USA) and *L. paracasei* OSU-PECh-3B from buttermilk produced by Dairy America (Fresno, CA, USA); two strains of *Lactobacillus plantarum* (*L. plantarum* OSU-PECh-A and *L. plantarum* OSU-PECh-BB) isolated from natural yogurt and pasteurized half and half, produced by Superior Dairy (Canton OH, USA); and two strains of *Pediococcus acidilactici* (*P. acidilactici* OSU-PECh-L) isolated from mozzarella cheese produced by Belgioioso (Green Bay, WI, USA) and (*P. acidilactici* OSU-PECh-3A) gouda cheese produced by Sargento (Plymounth, WI, USA).

### Localization of enzymatic activities in cell fractions

**Fig. 2:**
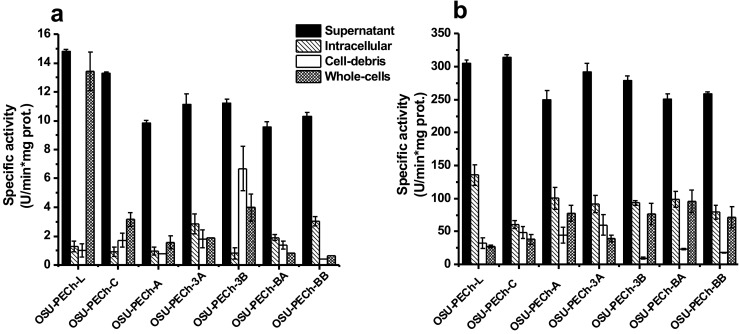
Cell localization of enzymatic activities. **a** Lipolytic activity using *p*-NPA as substrate; **b** proteolytic activity using azocasein as substrate. Error bars represent standard deviation of three independent experiments

After obtaining different cell fractions (supernatant, whole cells, intracellular, and cell-debris) by centrifugation and sonication from fresh cultures, respectively, the lipolytic, proteolytic, and antibacterial activities were evaluated. Figure 2a depicts lipolytic activity using *p*-NPA as the substrate. All seven strains exhibited the greatest hydrolysis of *p*-NPA in the cell supernatant relative to the rest of the fractions. OSU-PECh-L had the greatest lipolytic activity in both the supernatant and whole cell fractions compared with the other six strains and fractions. The OSU-PECh-3A and OSU-PECh-BB strains had the greatest hydrolysis values in the intracellular fraction relative to the other fractions of those strains. The cell-debris fraction from OSU-PECh-3B strain showed lipolytic activity (Fig. 2a).

Similar results were obtained for the proteolysis screening. The seven strains exhibited proteolytic activity in the supernatant fractions (Fig. 2b). The intracellular fraction of OSU-PECh-L also showed high hydrolysis of azocasein. Conversely, the cell-debris fraction of all examined strains had low proteolytic activity. The OSU-PECh-A, OSU-PECh-3B, OSU-PECh-BA, and OSU-PECh-BB strains had the greatest proteolysis in the whole cell fraction (Fig. 2b).

**Fig. 3:**
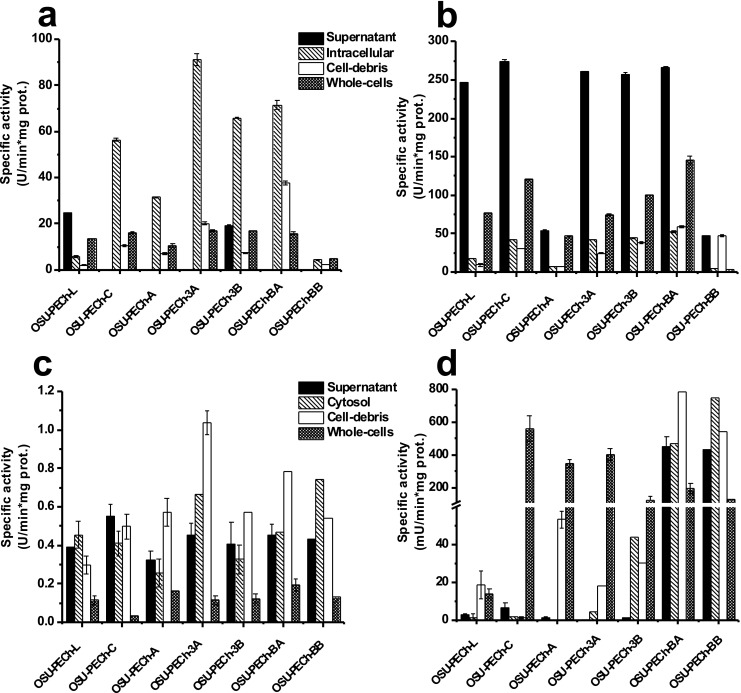
Cell localization of antibacterial activities. **a** N-acetylglucosaminidase; **b** N-acetylmuramoyl-L-alanine amidase; **c** N-acetylmuramidase; and **d** endopeptidase activity. Error bars represent standard deviation of three independent experiments

Antibacterial activity is shown in Fig. 3. Only two strains (OSU-PECh-L an OSU-PECh-3B) displayed hydrolysis in the supernatant fraction in the N-acetylglucosaminidase assay (Fig. 3a). The maximum values of N-acetylglucosaminidase activity was found in the intracellular fractions for four of the strains (OSU-PECh-C, OSU-PECh-3A, OSU-PECh-3B, and OSU-PECh-BA). The cell-debris fraction from OSU-PECh-BA had the highest activity against the substrate. The whole cell fractions from all strains also showed activity but was considerably lower compared with that obtained in the intracellular fraction. For the N-acetylmuramoyl-L-alanine amidase assay (Fig. 3b), the supernatant fraction showed values in five of the seven strains evaluated. Moreover, the whole cell fraction also showed activity in six of the seven strains, with OSU-PECh-C and OSU-PECh-BA showing the highest activities. The intracellular and cell-debris fractions had the lowest activity in all strains. Figure 3c depicts N-acetylmuramidase activity that hydrolyzes the cell wall of *M. lysodeikticus.* N-Acetylmuramidase activity was observed in all fractions and strains; however, the cell-debris fraction from OSU-PECh-3A had the highest activity value. Also, intracellular fraction from OSU-PECh-3A and OSU-PECh-BB showed high hydrolysis of *M. lysodeikticus*. Overall, the whole cell fraction from all strains revealed the lowest antibacterial activity against the cell wall of *M*. *lysodeikticus*. In terms of endopeptidase activity (Fig. 3d), only two strains (OSU-PECh-BA and OSU-PECh-BB) exhibited high hydrolysis of the substrate in the four fractions. The whole cell fraction exhibited endopeptidase activity in all 7seven strains, and OSU-PECh-L had the lowest activity, and the highest was OSU-PECh-C, among the bacteria tested.

In order to complement the aforementioned antibacterial activities, agar diffusion assays against pathogenic strains commonly found in dairy products were performed.

Table 1 shows the inhibition zone in mm/mg protein of each fraction of the seven LAB strains. The supernatant and whole cell fractions of the seven strains inhibited growth of *E. coli*, with the exception of the supernatant from the strain OSU-PECh-A and OSU-PECh-BA and the whole cells fraction from OSU-PECh-3B. None of the intracellular and cell-debris fractions showed inhibition of *E. coli*. The target strain *L. innocua* was inhibited by several strains and fractions. In general, all whole cell fractions had the ability to inhibit this microorganism. The supernatant from OSU-PECh-L showed high inhibition of *L. innocua*, and the supernatants of OSU-PECh-C and OSU-PECh-3B did not display antibacterial activity for *L. innocua*. Only three strains exhibited inhibition by the cell-debris fraction (OSU-PECh-A, OSU-PECh-3A and OSU-PECh-BB). On the other hand, *S. aureus* and *S. epidermidis* had similar patterns of inhibition between the strains, and these were inhibited by all fractions of OSU-PECh-BA and OSU-PECh-BB. The four fractions from OSU-PECh-C exhibited activity against *S. aureus*.

**Table 1:** Agar diffusion against pathogenic strains commonly found in dairy products (mm inhibition/mg protein)

Strain	Strain target	Supernatant	Intracellular	Cell-debris	Whole cells
*P*. *acidilactici* OSU-PECh-L	*E*. *coli*	58 ± 6	0	0	58 ± 5
	*L*. *innocua*	94 ± 3	49 ± 3	0	56 ± 4
	*S*. *aureus*	0	0	0	60 ± 3
	*S*. *epidermidis*	0	0	0	48 ± 4
*L*. *casei* OSU-PECh-C	*E*. *coli*	74 ± 3	0	0	62 ± 4
	*L*. *innocua*	0	0	0	92 ± 6
	*S*. *aureus*	69 ± 6	45 ± 8	56 ± 4	41 ± 4
	*S*. *epidermidis*	0	0	0	64 ± 7
*L. plantarum* OSU-PECh-A	*E*. *coli*	0	0	0	30 ± 4
	*L*. *innocua*	32 ± 7	0	30 ± 6	47 ± 4
	*S*. *aureus*	0	0	34 ± 6	48 ± 4
	*S*. *epidermidis*	44 ± 4	0	0	33 ± 3
*P. acidilactici* OSU-PECh-3A	*E*. *coli*	84 ± 4	0	0	66 ± 2
	*L*. *innocua*	69 ± 7	43 ± 8	48 ± 7	106 ± 5
	*S*. *aureus*	0	0	0	59 ± 7
	*S*. *epidermidis*	0	0	0	77 ± 3
*L*. *paracasei* OSU-PECh-3B	*E*. *coli*	68 ± 4	0	0	0
	*L. innocua*	0	0	0	60 ± 7
	*S*. *aureus*	0	55 ± 4	56 ± 7	65 ± 4
	*S. epidermidis*	0	53 ± 7	30 ± 6	93. ± 2
*L*. *paracasei* OSU-PECh-BA	*E. coli*	0	0	0	58 ± 4
	*L. innocua*	29 ± 4	56 ± 2	0	50 ± 4
	*S*. *aureus*	69 ± 4	77 ± 6	108 ± 6	106 ± 9
	*S*. *epidermidis*	100 ± 4	30 ± 6	56 ± 2	107 ± 6
*L*. *plantarum* OSU-PECh-BB	*E*. *coli*	67 ± 2	0	0	64 ± 3
	*L*. *innocua*	54 ± 5	0	86 ± 3	37 ± 3
	*S*. *aureus*	57 ± 4	78 ± 3	86 ± 3	99 ± 3
	*S*. *epidermidis*	86 ± 3	45 ± 8	68 ± 4	56 ± 2

### Identification and isolation of enzymes in zymograms

**Fig. 4:**
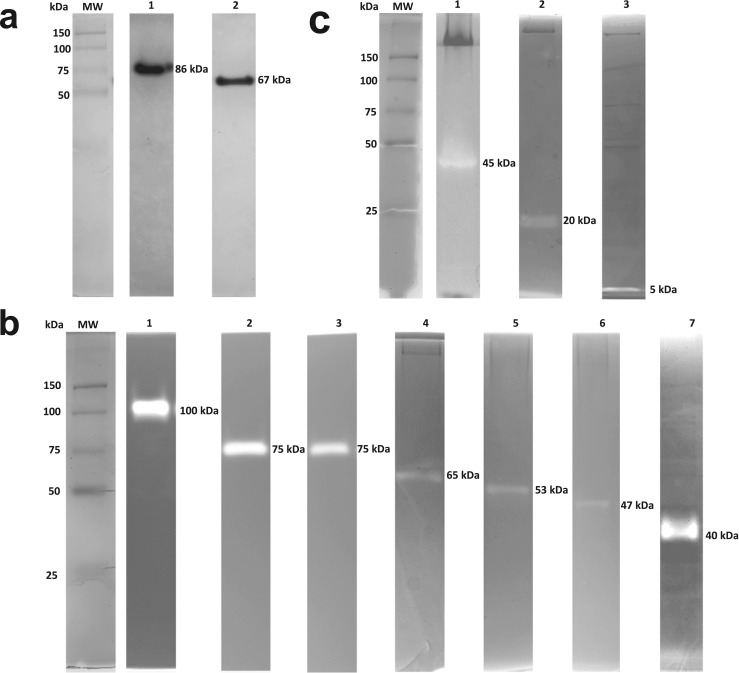
Zymograms without SDS- and SDS-PAGE. **a** Lipolytic activity; lane 1, intracellular fraction from OSU-PECh-3A; lane 2, intracellular fraction from OSU-PECh-C. **b** Proteolytic activity; lane 1, intracellular fraction from OSU-PECh-3B; lane 2, cell-debris fraction from OSU-PECh-C; lane 3, cell-debris fraction from OSU-PECh-3A; lane 4, intracellular fraction from OSU-PECh-BA; lane 5, intracellular fraction from OSU-PECh-C; lane 6, intracellular fraction from OSU-PECh-3B; lane 7, supernatant fraction from OSU-PECh-C. **c** Antibacterial activity; lane 1, supernatant fraction from OSU-PECh-3A; lane 2, supernatant fraction from OSU-PECh-3B; lane 3, supernatant fraction from OSU-PECh-3A. MW, molecular weight marker

Zymography was used to visualize the proteins responsible for lipolytic, proteolytic, and antibacterial activities in each fraction from the seven LAB strains. For lipolytic activity, native 10% PAGE was used to detected two proteins with experimental molecular weights of 86 and 67 kDa from the intracellular fractions of OSU-PECh-3A and OSU-PECh-C, respectively (Fig. 4a, lane 1 and 2). Lipolytic activity of these proteins was not observed under denaturing and reducing conditions (with the addition of SDS, DTT, and ß-mercaptoethanol, data not shown). To examine proteolytic enzymes, SDS-PAGE was used with casein as a substrate. The resulting gel led to the identification of seven different proteases (Fig. 4b). Species *L. casei* expressed three different proteases in the zymogram. One of these three proteins was located in the cell-debris fraction (75 kDa, Fig. 4b lane 2). The second protein, with a molecular weight of 53 kDa, was found in the intracellular fraction and the third protein (40 kDa) in the supernatant fraction (Fig. 4b, lanes 5 and 7, respectively). Three proteases were also observed in the intracellular fraction of *L. paracasei,* with molecular weights of 100, 65, and 47 kDa (Fig. 4b, lanes 1, 4, and 6, respectively). Lastly, one 75 kDa protease was found in the cell-debris fraction of *P. acidilactici* (Fig. 4b, lane 3). With regard to antibacterial activity, three proteins from the supernatant fraction with activity against *M. lysodeikticus* were detected. Two proteins were from OSU-PECh-3A strain with 45-kDa of molecular weight, and the second was observed in the bottom part of the SDS-PAGE with a 5 kDa molecular weight (Fig. 4c, lanes 1 and 2, respectively). The third antibacterial protein was observed in the OSU-PECh-3B strain with a 20 kDa of molecular weight.

### Amino acid sequence analysis

Amino acid sequence analysis identified 12 proteins with over 10% sequence identity to sequences reported in UniProtKB database. Table 2 shows the identity and putative functions of the different proteins identified by LC-MS/MS.

**Table 2:** Identification of proteins by LC-MS/MS from different fractions of LAB

Strain	Fraction	Protein function	No. peptides	% coverage	Access number	Theoretical MW (kDa)
Lipases						
*P*. *acidilactici* OSU-PECh-3A	Intracellular	Phosphoesterase	3	10	ARW28254.1	45.4
*L*. *casei* OSU-PECh-C	Intracellular	Esterase/lipase	4	17	CCK23986.1	35.0
Proteases						
*L*. *paracasei* OSU-PECh-3B	Intracellular	Peptidase M1	6	10	EEI69180.1	94.4
*L*. *casei* OSU-PECh-C	Cell-debris	Clp protease	17	33	CCK23115.1	77.9
*P*. *acidilactici* OSU-PECh-3A	Cell-debris	Clp protease	13	25	WP_070366152.1	77.9
*L*. *paracasei* OSU-PECh-BA	Intracellular	Neutral endopeptidase	6	13	WP_016379304.1	71.3
*L*. *casei* OSU-PECh-C	Intracellular	Oligoendopeptidase	4	12	CCK22105.1	67.4
*L*. *paracasei* OSU-PECh-3B	Intracellular	Aminopeptidase C	6	28	KRM98411.1	50.5
*L*. *casei* OSU-PECh-C	Supernatant	Serine protease	4	12	WP_071798966.1	44.9
Antibacterial proteins						
*P*. *acidilactici* OSU-PECh-3A	Supernatant	N-acetylmuramidase	4	14	WP_075139824	48.0
*L*. *paracasei* OSU-PECh-3B	Supernatant	Hydrolase-amidase	3	18	KRN07451	22.8
*P*. *acidilactici* OSU-PECh-3A	Supernatant	50S ribosomal protein	2	69	WP_002832248.1	4.4

#### Lipolytic proteins

Amino acid sequence analysis identified two proteins with esterase/lipase activity. The first was isolated from the intracellular fraction of OSU-PECh-3A with an observed molecular weight around 86 kDa in a native zymogram (Fig. 4-A, lane 1). Trypsin digestion of this protein generated 3 peptides that matched a 45.5 kDa putative metallophosphoesterase from the *P. acidilactici* SRCM101189 genome. The difference in molecular weight suggests that the active oligomeric form is a dimer. This enzyme is part of a metal-dependent superfamily containing a conserved domain in the active site with histidine, aspartate, and asparagine residues. The second lipolytic protein, isolated from OSU-PECh-C, was a 67 kDa protein as observed in the native zymogram (Fig. 4-A, lane 2). It was identified as a 35 kDa esterase/lipase, suggesting that it is also found as a dimer in the active form. By definition, esterases and lipases hydrolyze the carboxylic ester bond of both short and long-chain fatty acids.

#### Proteolytic enzymes

Proteases were the most abundant polypeptides found in the bacterial cell fractions. The cellular location and molecular weights of 7 different proteins, each cleaving at specific amino acid residues, were identified. Three of these proteases were found in the *L. casei* genome, the first one produced 17 peptides after trypsin digestion and matched a 77.9 kDa putative Clp protease (33% coverage). This ATP-dependent protease is a component of the Clp chaperone-protease complex that is involved in protein degradation and disaggregation. Another protein, identified by 4 peptides after digestion, was an oligoendopeptidase with a molecular weight of 67 kDa. This protein belongs to the thermolysin-like family of proteinases and zinc-dependent metallopeptidases and is widely used as a nonspecific protease to degrade extracellular proteins and peptides for bacterial nutrition. The last *L. casei* protease (identified by 4 peptides) was a serine protease with a putative molecular weight of 44.9 kDa. This protease cleaves peptide bonds using a specific set of amino acid residues in the active site, one of which is always a serine residue. In *L. paracasei* genus*,* 3 proteins were identified in the intracellular fraction. The first protease (identified by 6 peptides) is part of the Peptidase M1 family from the *L. paracasei* 25,302 genome and has a putative molecular weight of 94.4 kDa. This protein is from the aminopeptidases-N and glutamyl-aminopeptidase group, which release N-terminal glutamate from peptides. Six peptides were produced after trypsin digestion of the second protease, which was identified as a 71.3 kDa neutral endopeptidase from the *L. paracasei* genome. This protein is from the peptidase M13 family and is used to digest milk proteins. The third protein (identified by 6 peptides after digestion) was a 50.5 kDa aminopeptidase C from *L. paracasei* DSM 20258. This exopeptidase is a cysteine peptidase that hydrolyses a peptide bond using the thiol-group active site of a cysteine residue. Interestingly, the complete sequences of these three enzymes from *L. paracasei* strains did not show similarity between one another, settling that are different proteins (data not shown; MUSCLE, multiple sequence comparison by log-expectation).

Finally, a protease in the intracellular fraction of *P. acidilactici* OSU-PECh-3A was identified by 13 peptides from the trypsin digestion. This protein resembles an ATP-dependent Clp protease with molecular weight of 77.9 kDa (25% coverage). It had similar activity and a similar molecular weight to the *L. casei* OSU-PECh-C protease. However, multiple alignment between these proteases did not show similarity in amino acid sequence (data not shown).

#### Antibacterial proteins

Lastly, three proteins with antibacterial activity were identified from the supernatant fractions. One of these belongs to the strain OSU-PECh-3A and was identified as a 48 kDa N-acetylmuramidase using 4 peptides, which accounted for 14% coverage of the sequence. This protein is a peptidoglycan hydrolase that degrades bacterial cell walls through hydrolysis of 1,4-β-linkages between N-acetylmuramic acid and N-acetylglucosamine residues. The second protein, found in OSU-PECh-3B, had a molecular weight of 20 kDa and was digested into 3 peptides that represented 17.8% coverage. The protein was identified as a hydrolase-amidase with theoretical molecular weight of 22.8 kDa. This protein is structurally related to other amidohydrolases (i.e. N-acetylmuramoyl-L-alanine amidase) that are involved in the degradation of the peptidoglycan and hydrolysis of the amide bond between N-acetylmuramic acid and L-amino acids of the bacterial cell wall. The last protein found was the 50S ribosomal protein L36, which was identified by 2 peptides after digestion and matches 69% of the total sequence of this protein from the *P. acidilactici* 7_4 genome. This protein has an experimental molecular weight of 5 kDa and was found in the supernatant fraction of OSU-PECh-3A.

## Discussion

LAB contribute to the flavor characteristics of fermented food products as a result of fat, protein, and carbohydrate breakdown. Lipolysis and proteolysis are significant in the fermentative processes of dairy products because the hydrolysis of triacylglycerols and proteins in milk produce favorable compounds. Previous studies have failed to consider both lipolytic and proteolytic activities concurrently. Some studies focusing on other fermented products, such as wine or meat, screened for either the lipolytic or proteolytic activity of bacteria (Kliche et al. 2017; Pérez-Martín et al. 2013). In this study, 137 strains isolated from dairy products were screened for both lipolytic and proteolytic activities. Seven strains displayed both high lipolytic and proteolytic activities. In the proteolysis experiments, the positive control (*L. lactis*) showed the greatest activity, however, some isolated strains also exhibited high activity values. Lipolysis was greatest in the isolated strains compared with the positive control. *L. lactis* was selected as the positive control because it is one of the most studied microorganisms in dairy products, it generates volatile compounds from the hydrolysis of milk fat, and it is capable of efficiently hydrolyzing milk proteins (Dhaisne et al. 2013). The seven strains with high lipolytic and proteolytic activities were selected and further examined with the intention of understanding the role of lactic acid bacteria during fermentation of dairy products and whether potentially healthful metabolites could be produced. These seven strains were genetically identified as: *L. casei*, two strains of *L. paracasei*, two strains of *L. plantarum*, and two strains of *P. acidilactici*.

Five of the seven strains isolated belong to the *Lactobacillus* genus, which has been generally characterized as lipase-producing. For example, lipases have been cloned, expressed, purified, and characterized in *Lactobacillus plantarum*, *Lactococcus lactis*, and *Lactobacillus casei*. These enzymes have since been applied to dairy products and have displayed a positive effect on the organoleptic proprieties of these foods (Esteban-Torres et al. 2014; Fernandez et al. 2000; Castillo et al. 1999). Another genus, *Pediococcus*, has been shown to be lipolytic; however, lipolytic activity of this genus is more frequently reported in the fermentation of grape must, wine, and meat products (Pérez-Martín et al. 2013; Vaquero et al. 2004; Ostdal et al. 1996). In this study, two *P. acidilactici* strains with high lipolytic activity were isolated from dairy products, which have the potential to influence the aromatic compounds and the sensory characteristics of the fermented foods. On the other hand, proteolytic activity has been reported several times for LAB, including *Enterococcus*, *Lactobacillus*, *Lactococcus*, and *Pediococcus*. In this work, 84 of 137 strains isolated had proteolytic activity, corresponding to 61.3% of the total isolates. This indicates that more than half of the LAB isolated from these dairy products have proteolytic activity and likely a direct effect on sensory properties, such as flavor formation and aroma in ripening foods (Simitsopoulou et al. 1997). The hydrolysis of proteins in milk (i.e., casein, ß-lactoglobulin, and α-lactalbumin) produces free amino acids and small peptides, which have been suggested to exhibit anti-inflammatory and antioxidant effects in humans (Kliche et al. 2017). Furthermore, these hydrolyzed peptides are beneficial to gut microbiota because they show antimicrobial activity and inhibit pathogenic microorganisms such as *E. coli*, *L. monocytogenes*, and *S. aureus*, thereby promoting the growth of LAB (Mohanty et al. 2016).

Out of all LAB, the proteolytic system of *Lactococcus lactis* is the most extensively studied in terms of regulation and hydrolysis of milk proteins as casein (Savijoki et al. 2006). Also, the *Lactobacillus* genus, specifically *L. delbrueckii* and *L. helveticus*, produce proteases that can hydrolyze α- and ß-casein (Kliche et al. 2017). The *Pediococcus* genus displays high levels of proteolysis and is commonly found in meat and cheese products (Gandhi et al. 2016; Simitsopoulou et al. 1997). In this study, only two of the selected seven strains belong to the *Pediococcus* genus, while the other five strains belong to the *Lactobacillus* genus indicating a large range of species and characteristics of LAB found in dairy products.

Through investigating the subcellular localization and relative solubility of these enzymes, the processes involved in ripening and fermentation of dairy and meat products are becoming better understood. In this study, four subcellular fractions (the supernatant, intracellular, cell-debris, and whole cell fractions) were compared with understand the organization of the lipolytic, proteolytic, and antibacterial activity in LAB. The relationship between the location of these enzymes and their type of activity adds insight to their roles during dairy fermentation. This work provides evidence that lipolytic and proteolytic activities are apparent in the supernatant fraction, meaning these enzymes are released into extracellular space. Together, this suggests that these enzymes are released into food matrices where they hydrolyze milk fat and proteins, thus, having a direct effect on flavor development and the biochemistry of dairy products. In correspondence with these findings, other authors have reported these activities in the supernatant fractions. For example, the extracellular fractions of *L. acidophilus* O177 and *L. mesenteroides* subsp. *dextranicum* O257 hydrolyzed tributyrin and buttermilk (Katz et al. 2002). In addition, the extracellular proteolytic system of LAB plays a key role in degrading casein into oligopeptides in milk medium, which can then utilize in their metabolic processes (Savijoki et al. 2006).

Some authors have also reported these activities in cell-free extracts (intracellular), implying the rupture of cells and release of intracellular enzymes into the matrix (Hong et al. 2018; Abeijon-Mukdsi et al. 2009; Sumby et al. 2009). The other fractions (cell-debris, supernatant, and whole cells) showed lower levels of lipolysis and proteolysis, suggesting less of these enzymes are present in these fractions and their respective strains that improve the organoleptic properties of dairy products (Pérez-Martín et al. 2013).

Antibacterial activity, such as N-acetylglucosaminidase activity, is commonly found in the extracellular fraction which could pose multiple health benefits (Jankovic et al. 2010). In this study, two strains had extracellular N-acetylglucosaminidase activity. This enzyme degrades bacterial cell walls by catalyzing the hydrolysis of terminal, non-reducing N-acetylglucosamine residues from oligosaccharides. It is believed that N-acetylglucosaminidase activity could be beneficial to human health because it can process and metabolize free oligosaccharides in human milk. These products then play an important role in establishing and maintaining the infant gut microbiome while reducing non-beneficial bacteria (Bidart et al. 2015). In addition, this study provided evidence of N-acetylglucosaminidase activity intracellularly, where it is not commonly reported.

Antibacterial activity via N-acetylmuramoyl-L-alanine amidase was also examined and found mostly in the extracellular fractions. N-acetylmuramoyl-L-alanine amidase is significant to dairy products because it hydrolyzes the bond between the glycan polymer and peptide in peptidoglycan to destabilize it and release intracellular enzymes involved in cheese ripening and flavor characteristics (Jebava et al. 2011; Moscoso and Suárez 2000). Whole cells also showed activity, indicating that surface proteins on cell can hydrolyze amide bonds of peptidoglycan and release free amino groups, contributing to flavor and aroma of dairy products and as well the innocuity of the final product (Lortal and Chapot-Chartier 2005). There are many reports of N-acetylmuramidase activity as an effective antibacterial agent, which cleaves the glycan bond of peptidoglycan in bacterial cell walls. This activity was found in supernatant, intracellular, and cell-debris fractions in this study and probably plays an important role in dairy processing, because it promotes flavor development by releasing intracellular compounds, such as lipases, proteases, and amino group. Additionally, it inhibits some microorganisms, decreasing the prevalence of undesirable bacteria in the product (Pang et al. 2014). N-acetylmuramidase activity was also reported in transgenic cattle milk to prevent mastitis in vitro (Yang et al. 2011). On the other hand, this study detected two strains with high activity against *S. aureus* cells in all fractions, indicating endopeptidase activity. This endopeptidase cleaves peptide bonds between two amino acids, especially in bacterial cell walls. Therefore, these strains can be used in milk or milk products to reduce the infection risk by *S. aureus*. Mitigation of this microorganism is significant because it causes both systematic and ocular infections in humans and acute mastitis in cows (Verbree et al. 2017). Additionally, after cell hydrolysis, intracellular compounds are released, which affect the organoleptic characteristics in dairy products. In order to complement the antibacterial activities, agar diffusion showed broad variation in inhibition of pathogenic strains commonly found throughout dairy processing. For example, pathogenic *E. coli* has a prevalence of approximately 0.9% in dairy products and causes foodborne diseases, like hemolytic uremic syndrome, diarrhea, and possibly death (Douëllou et al. 2017). This study found six strains with antibacterial activity against *E. coli*, which makes them promising starter culture strains in products with a high risk of *E. coli* contamination. On the other hand, the *Listeria* genus is frequently reported as pathogenic, and the prevalence in dairy products as yogurt, ice cream, and cheese are common (Rocourt and Buchrieser 2007). In this work, all bacteria and different fractions evaluated had a high activity against *L. innocua*, which indicates that the LAB studied can minimize the presence of this species in dairy products. In future studies, it would be interesting to evaluate the effectiveness against other pathogenic strains, such as *L. monocytogenes* and *L. ivanovii* that are of public health significance (Evert-Arriagada et al. 2018). *S*. *aureus* and *S*. *epidermidis* are the most contagious strains that produce bovine mastitis. For several years, milk producers, governments, and researchers have tried to reduce contamination by these microorganisms (Sunagar et al. 2013). In this study, three strains (and all the fractions of these strains) of LAB examined inhibited *Staphylococcus* genus, which suggests that the use of LAB in dairy products has a promising future for the reduction of these microorganisms.

A protein with lipolytic activity was detected by zymography in the intracellular fraction of *P. acidilactici.* This is the first lipase reported for the *Pediococcus* genus isolated from a dairy product, which under native PAGE had a molecular weight of 86 kDa. The peptides found by LC-MS/MS indicate it is a putative metallophosphoesterase with a theoretical molecular weight of 45.5 kDa, suggesting that this protein is active as a homodimer. This protein has been previously reported in another microorganism, *L. plantarum* 2739. Gobbetti et al. (1996) found this protein in the intracellular fraction with a molecular weight of 85 kDa, however, the first 15 N-terminal amino acids had no similarity with the sequence reported for the intracellular esterase in *P. acidilactici* isolated in this study (data not shown; MUSCLE, multiple sequence comparison by log-expectation).

Several proteolytic enzymes identified by LC-MS/MS were observed in different cell fractions. The molecular weights detected by zymography were between 40- and 100-kDa, indicating a large range of proteases that LAB produce. Proteolysis is a biochemical process important in the maturation or fermentation of dairy products. As mentioned above, the proteolytic system of LAB is well studied and has been classified as extracellular, membrane, and intracellular proteins. In general, casein hydrolysis begins with a cell-envelope proteinase that cleaves the protein into smaller peptides (oligopeptides). These oligopeptides are then transported into bacteria by other membrane transporters and peptidases that degrade the oligopeptides into amino acids (Savijoki et al. 2006).

In this study, proteases in the supernatant, membrane, and within the cell were detected and sequenced suggesting that other genera and species besides *Lactococcus lactis* show a similar proteolytic system to that described above. On the other hand, the seven identified proteins are reported as putative and have not been natively characterized.

Finally, two proteins with antibacterial activity and a third with suspected bacteriocin activity were identified. These are putative proteins and have not been reported so far. The first protein was identified from *P. acidilactici* with a 45 kDa molecular weight by zymograms found in the supernatant fraction. The database reports that this protein has N-acetylmuramidase activity. Although this genus is generally considered a bacteriocin-producer, recent reports show that *Pediococcus* is not exclusive to bacteriocin production and can present N-acetylmuramidase, N-acetylmuramoyl-L-alanine amidase, or N-acetylglucosaminidase activities against a broad spectrum of pathogenic bacteria found in food (Mora et al. 2003; García-Cano et al. 2015). The second protein is a hydrolase-amidase found in the supernatant fraction of *L. paracasei* with a theoretical molecular weight of 22.8 kDa. This 20 KDa protein showed lytic activity against *M*. *lysodeikticus*. The hydrolase-amidase protein family has been reported as highly active against *S. aureus* biofilms, significantly decreasing infections by this microorganism (Schmelcher et al. 2015). Several reports describe the antibacterial activities against foodborne pathogens found in bovine milk and dairy products. These enzymes have been used directly in bovine milk to decrease the presence of *S. aureus* and prevent the unwanted gas formation during cheese ripening (Schmelcher and Loessner 2016). The third antibacterial protein identified was a 5 kDa 50S ribosomal protein from *P. acidilactici*. At first, these low molecular weight proteins were believed to be a type of bacteriocin, called a pediocin, which also has a low molecular weight (Mora et al. 2003). More recently, these low molecular weight proteins have been characterized in other species and identified as ribosomal proteins such as 30S or 50S. For example, the low molecular weight antibacterial protein sakacin P (4.4 kDa) and a 30S ribosomal protein (6.8 kDa) both found in *L. sakei* have been reported with activity against *L*. *monocytogenes* (de Carvalho et al. 2010). Two 50S ribosomal proteins (11 and 6.5 kDa) with non-bacteriocin antibacterial activity in *L*. *salivarius* are also proteins with low molecular weight with antibacterial activity (Pidutti et al. 2018). These low molecular weight proteins can be classified as a new group of peptides with antibacterial activity. They can be used in foods in conjunction with other inhibitory proteins, such as those found in this project. By incorporating both these low molecular weight peptides and other antimicrobial proteins (each with different mechanisms of action) into dairy products, it is hypothesized that a synergistic antimicrobial effect against pathogenic bacteria will be produced and reduce the risk of some foodborne diseases.

Originally, it was suspected that LAB have the same or similar enzymatic activities. Despite these expectations, the bacteria studied in this work have unique activity profiles, showing promise for in vitro and in vivo applications. These bacteria have the potential add value to the safety and sensory attributes of dairy products, thus, presenting dual activities that favor the metabolism of lipids and proteins found in milk. The ability of different LAB to coexist in a single food implies the potential to use a selected consortium of bacteria with high values of enzymatic activities in the development of foods to promote consumer health.
